# Variation of Carbohydrate-Active Enzyme Patterns in the Gut Microbiota of Italian Healthy Subjects and Type 2 Diabetes Patients

**DOI:** 10.3389/fmicb.2017.02079

**Published:** 2017-10-24

**Authors:** Matteo Soverini, Silvia Turroni, Elena Biagi, Sara Quercia, Patrizia Brigidi, Marco Candela, Simone Rampelli

**Affiliations:** Unit of Microbial Ecology of Health, Department of Pharmacy and Biotechnology, University of Bologna, Bologna, Italy

**Keywords:** gut microbiota, microbiota-accessible carbohydrates, CAZymes, co-abundance groups, type 2 diabetes

## Abstract

The human gut microbiota (GM) has been associated, to date, with various complex functions, essentials for the host health. Among these, it is certainly worth noting the degradation of the so-called microbiota-accessible carbohydrates (MACs), which the GM breaks down through specific enzymes, referred to as carbohydrate-active enzymes (CAZymes). This degradation constitutes the first step in the production of short-chain fatty acids (SCFAs), key microbial small molecules having multiple health-promoting effects for the host organism. The decline in MAC dietary intake in urban Western populations forced the shrinkage of CAZyme repertoire in the GM, as shown by the literature comparing the microbiome layout between Western urban citizens and traditional rural populations. Even if this reduction in GM functional complexity has been associated with the onset of the so-called “diseases of civilization,” only few information regarding the CAZyme variation within Western populations has been provided to date, and its connections with diet and health are still unexplored. In this scenario, here we explore the GM-encoded CAZyme repertoire across two Italian adult cohorts, including healthy lean subjects consuming a Mediterranean diet and obese patients affected by type 2 diabetes, consuming a high-fat diet. In order to impute the CAZyme panel, a pipeline consisting of publicly available software – QIIME, FragGeneScan and HMMER – was specifically implemented. Our study highlighted the existence of robust clusters of bacterial species sharing a common MAC degradation profile in the Italian GM, allowing the stratification of the individual GM into different steady states according to the carbohydrate degradation profile, with possible connections with diet and health.

## Introduction

The large ensemble of bacteria that stably inhabit the distal part of our gastrointestinal tract, namely the gut microbiota (GM), is of vital importance for many physiological functions of our organism, exerting a key role in several biological processes, such as nutrition, immune function, and the regulation of the central nervous system (Neish, 2009; Hooper et al., 2012; Nicholson et al., 2012; Honda and Littman, 2016; Sharon et al., 2016). As a strategic partner of the human holobiont, the GM expands our metabolic potential by adding degradative functions, thus enabling the metabolism of a wide range of complex polysaccharides, otherwise indigestible, including exogenous carbohydrates introduced with diet (Schnorr et al., 2014). Indeed, despite the wide distribution of complex polysaccharides in edible plants (Lattimer and Haub, 2010), our genome is astonishingly poor in genes coding for carbohydrate-active enzymes (CAZymes) (Gill et al., 2006), i.e., enzymes specifically devoted to polysaccharide degradation. Conversely, the gut microbiome (i.e., the cumulative genome of the GM) encodes for a much wider and diversified arsenal of CAZymes (El Kaoutari et al., 2013), allowing for the catabolism of the vast array of complex polysaccharides that reach the colon undigested. These molecules are converted to short-chain fatty acids (SCFAs), microbial metabolites with multiple roles in human physiology (Koh et al., 2016). This inter-kingdom cross-feeding is the basis of the mutualistic relationship we share with our intestinal microbial counterpart, which thus emerges as a key player in the co-metabolism of complex carbohydrates in our gut. In this scenario, the dietary polysaccharides that are metabolically available to gut microbes have been specifically defined as microbiota-accessible carbohydrates (MACs) (Sonnenburg and Sonnenburg, 2014) and their abundance in the host diet, and consequently their availability within the gut, has proved to be crucial for microbiota–host homeostasis.

The MAC availability in the host diet is among the aspects that have deeply changed in recent human evolutionary history, with the transition from Paleolithic hunting-gathering to Neolithic rural populations and contemporary Westernized societies (Quercia et al., 2014; Schnorr et al., 2014; Sonnenburg and Sonnenburg, 2014; Obregon-Tito et al., 2015). In particular, along with Westernization, we have witnessed a progressive reduction of the MAC content and diversity in the diet, with the transition to foods high in refined simple sugars. According to Sonnenburg et al. (2016), this reduction of dietary MACs forced the progressive impoverishment of the CAZyme repertoire in the human microbiome, compromising the overall metabolic plasticity of the human meta-organism. This was confirmed by gut metagenome studies comparing the CAZyme repertoire between traditional populations consuming high-fiber diets and Western urban citizens, which highlighted a relevant reduction of the microbial CAZyme diversity in the latter (King et al., 2012; Bhattacharya et al., 2015; Rampelli et al., 2015). Such wide GM comparative surveys provided important information on the GM-host co-evolutionary dynamics but they did not allow for a precise discrimination of the impact of individual covariates (e.g., diet, health, medication) on the GM functional repertoire. Aiming to fill this gap in knowledge, here we compared the imputed CAZyme repertoire from two cohorts of Western urban adults from Italy, overcoming the intrinsic variation of the CAZyme profiles according to geography and ethnicity (Soverini et al., 2016). In particular, we analyzed the GM structure and metadata from16 healthy subjects following a Mediterranean diet and 40 obese, type 2 diabetic (T2D) patients consuming a high-fat low-MACs diet from two previously published studies (Schnorr et al., 2014; Candela et al., 2016). Our findings highlighted the existence of a potentially limited number of well-balanced host–microbe symbiotic configurations, with a possible connection to diet and health status.

## Materials and Methods

### Determination of the Pan-microbiome from Italian Healthy Subjects

The publicly available 16S rRNA sequencing data of the fecal samples of 16 Italian healthy subjects from Schnorr et al. (2014) were downloaded from the MG-RAST website^1^ and taxonomically characterized to the species level using the QIIME pipeline (Caporaso et al., 2010), with blastn as an assignment method and the HMP gastrointestinal 16S rRNA dataset as reference sequences^2^. The detected species were considered part of the so-called Italian “pan-microbiome,” i.e., the virtual entity gathering the vast majority of bacterial species present in the GM of the Italian population. The assembled reference genomes of these bacterial species were downloaded from the NCBI genome section^3^. Then, to characterize the CAZyme repertoire of these microorganisms, the CAZyme identification pipeline developed by Soverini et al. (2016) was applied. Briefly, ORFs were extracted from the assembled genomes using FragGeneScan 1.16 (Rho et al., 2010). From the translated ORFs, the CAZyme-coding sequences were detected using the hmmscan tool of the HMMER software package (Robert et al., 2011) and the dbCAN CAZyme database (Yin et al., 2012). The outputs were further processed by the script hmmscan-parser.sh^4^, selecting only the ORFs that showed a minimum identity of 30% to the query sequences and an alignment length of at least 100 residues.

### Identification of CAZyme Co-abundance Groups within the Italian Pan-microbiome

The CAZyme profiles were used to generate CAZy co-abundance groups (CCGs), which were conceived as groups of bacterial species sharing a similar CAZyme profile. In brief, the CCGs were generated by applying hierarchical Ward-linkage clustering based on Spearman correlation coefficients to the abundances of glycosyl-hydrolase (GH) and auxiliary activity (AA) families detected in the bacterial genomes. Permutational multivariate analysis of variance (function “adonis” of the vegan package in R) was used to determine whether CCGs were significantly different from each other. CAZymes were also manually classified for their ability to degrade specific substrates by consulting the publicly available CAZy database^5^. Specifically, we evaluated the ability to degrade different types of MACs: resistant starch (RS), non-digestible carbohydrates (NDC), non-starch polysaccharides (NSP), and mucins/glycoproteins (M/G). When more than one activity was found, we selected the most relevant one, i.e., the one with the highest abundance of genes involved in the degradation of a given substrate.

### Assessment of Redundant Patterns of CAZymes in Italian Healthy Subjects and Type 2 Diabetes Patients

To explore CAZyme profiles in the Italian population in health and disease, we integrated the dataset used to determine the pan-microbiome with the 16S rRNA sequences of the GM from 40 patients affected by type 2 diabetes (Candela et al., 2016). The sequences were downloaded from MG-RAST^6^ and analyzed using QIIME (Caporaso et al., 2010) and the HMP database, as described above for healthy subjects. The CAZyme profile of each GM was obtained by quantifying the relative abundance of each CCG, as a sum of the relative contribution of component bacterial species. We then grouped the subjects using hierarchical Ward-linkage clustering based on Spearman correlation coefficients. Separation between clusters was tested using the permutational multivariate analysis of variance (function “adonis” of vegan). All statistical analyses were computed in R version 3.1.3 using R studio version 1.0.36 with packages vegan and made4.

## Results

### The Italian Pan-microbiome from Healthy Adults

The analysis of the publicly available 16S rRNA sequences of stool samples from 16 Italian healthy adults (aged 20–40 years, mean 32 years) consuming a standard Mediterranean diet (Schnorr et al., 2014) led to the identification of a total of 98 bacterial species, present at least once in the samples (**Supplementary Table S1**). *Faecalibacterium prausnitzii* was the most represented species, with an average relative abundance of 11 ± 0.09% (standard deviation of the mean). *Eubacterium rectale* (7 ± 0.06%), *Ruminococcus bromii* (6 ± 0.05%), and *Bifidobacterium adolescentis* (6 ± 0.06%) occurred as co-dominant species. *Subdoligranulum variabile* (3 ± 0.04%), *Ruminococcus champanellensis* (3 ± 0.02%), *Clostridium asparagiforme* (2 ± 0.01%), *Bacteroides vulgatus* (2 ± 0.03%), *Coprococcus eutactus* (2 ± 0.03%), and *Roseburia intestinalis* (1.8 ± 0.01%) were ancillary species present to a lower extent. In terms of prevalence, *F. prausnitzii, E. rectale*, *Butyricicoccus pullicaecorum, R. intestinalis, Ruminococcus* spp*., Flavonifractor plautii, Blautia obeum, Dorea formicigenerans* and *Anaerostipes hadrus* were present in all samples of the dataset, whereas *Methanobrevibacter smithii, Bifidobacterium catenulatum, Lactobacillus ruminis, Weissella paramesenteroides*, and *Sutterella parvirubra* were found in less than three samples, being among the least prevalent microorganisms in the analyzed Italian microbiomes.

### CAZyme Repertoire in the Bacterial Species of the Italian Pan-microbiome

Reference genomes from the bacterial species included in the Italian pan-microbiome were retrieved from the NCBI database, and the respective CAZyme-coding sequences were identified. The number of these sequences varied widely between the pan-microbiome components. In particular, *Odoribacter laneus*, *Lactobacillus salivarius*, and *F. plautii* showed the lowest amounts of CAZyme-coding genes, while *Eubacterium biforme*, *Dialister succinatiphilus*, and *D. formicigenerans* the highest (see Supplementary Figure S1 for retrieving the number of CAZyme-coding sequences for each bacterial species).

**FIGURE 1:**
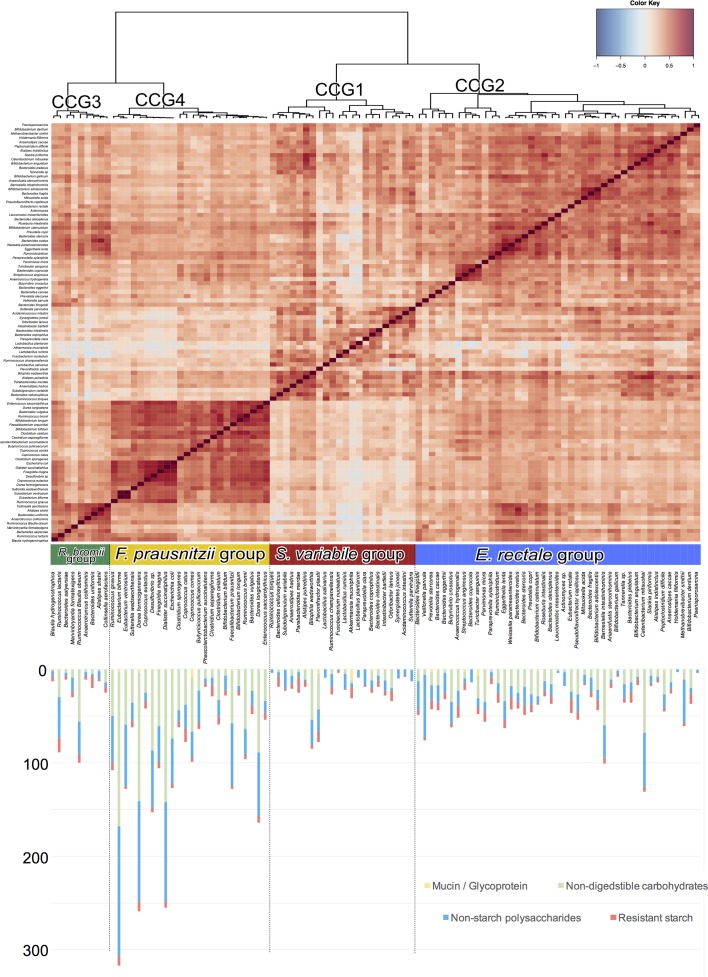
Hierarchical clustering of the raw abundances of the CAZy glycosyl-hydrolase (GH) and auxiliary activity (AA) families in the bacterial species constituting the Italian pan-microbiome. Spearman distance and Ward’s minimum variance method were used. Four CAZy Co-abundance Groups (CCGs) were identified and named according to the most abundant species in each group, as follows: CCG1 – *Subdoligranulum variabile* group (red), CCG2 – *Eubacterium rectale* group (blue), CCG3 – *Ruminococcus bromii* group (green), and CCG4 – *Faecalibacterium prausnitzii* group (yellow). Below the heatplot are reported the counts of CAZyme-coding sequences belonging to the GH and AA families, detected in the type strain reference genomes for each bacterial species, divided by class of MACs: resistant starch (RS), non-digestible carbohydrates (NDC), non-starch polysaccharides (NSP), and mucins/glycoproteins (M/G).

To identify common patterns of MAC-degrading enzymes among the different species of the Italian pan-microbiome, we determined co-abundance associations between the bacterial CAZyme profiles and then clustered them based on similarity. Four robust CAZy Co-abundance Groups (CCGs) were identified in the Italian pan-microbiome, each one including GM species that share a similar CAZyme profile, describing the pattern of CAZyme variation within the Italian GM species (*p*-value < 0.001, permutation test with pseudo *F* ratios). CCGs were named according to the most abundant species in each group as follows: *S. variabile* (CCG1), *E. rectale* (CCG2), *R. bromii* (CCG3), and *F. prausnitzii* (CCG4) (**Figure 1**). Interestingly, *F. prausnitzii* and *R. bromii* CCGs included species with the highest amount of CAZyme-coding sequences, such as *D. succinatiphilus*, *D. formicigenerans*, and *E. biforme*. On the other hand, CCG1 (*S. variabile*) and CCG2 (*E. rectale*) included species with a lower amount of CAZyme genes, such as *O. laneus* and *F. plautii*. It is important to note that the bacterial genome size is not predictive of the number of encoded CAZymes (El Kaoutari et al., 2013). For example, *Bacteroides oleiciplenus*, *Bacterioides cellulosilyticus* and *Bacteroides ovatus*, the three species with the largest genome size in the Italian pan-microbiome (Supplementary Figure S1), were found to be included within the *S. variabile* group and show the lowest richness in the CAZyme repertoire.

**FIGURE 2:**
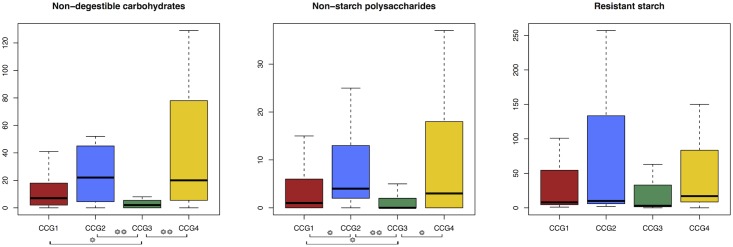
Boxplots representing the distribution of the degradative potential of exogenous MACs in the different CAZyme Co-abundance Groups (CCGs). Square brackets at the bottom indicate a significant difference in raw abundances (single asterisk, *p*-values between 0.05 and 0.001; double asterisk, *p*-values below 0.001, Wilcoxon rank sum test).

Finally, we specifically evaluated the distribution of CAZyme families involved in the degradation of different types of MACs across the four CCGs (**Figure 2**). According to our findings, *E. rectale* and *F. prausnitzii* groups showed the highest number of sequences encoding for CAZymes involved in the degradation of non-digestible carbohydrates and non-starch polysaccharides, while the *R. bromii* group was the least rich in these enzymatic functions (*p*-value < 0.05, Wilcoxon rank sum test). Interestingly, the ability to degrade RS was evenly spread among the different CCGs.

### CAZyme Distribution in the Gut Microbiome of Italian Individuals: Associations with the Diet and Health Status

**FIGURE 3:**
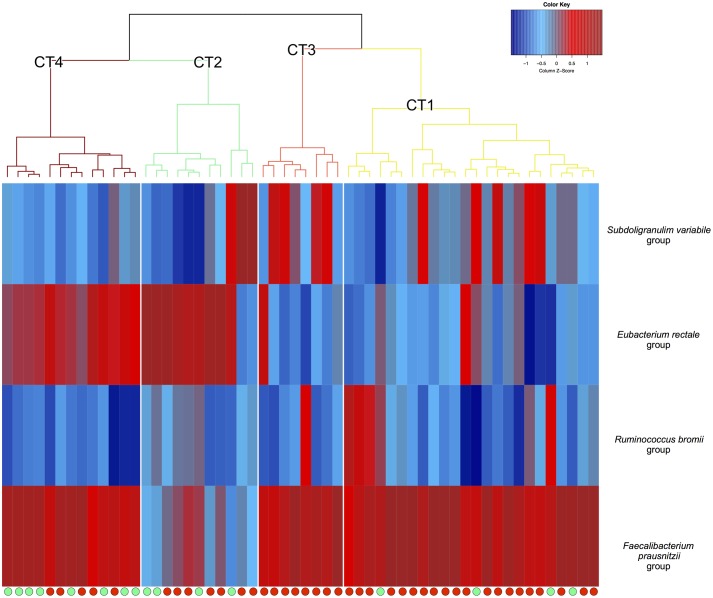
Hierarchical clustering of the relative abundances of each CAZyme Co-abundance Group (CCG) in the gut microbiota (GM) of every subject. Bray–Curtis distance and Ward’s minimum variance method were used. On the top: the four CAZyTypes (from CT1 to CT4) identified, i.e., clusters of different GM configurations with a similar carbohydrate-degrading profile. On the right: the four CCGs, named according to the most abundant species in each group (CCG1 – *Subdoligranulum variabile* group, CCG2 – *Eubacterium rectale* group, CCG3 – *Ruminococcus bromii* group, and CCG4 – *Faecalibacterium prausnitzii* group). At the bottom: green dot, healthy lean subject; red dot, obese type 2 diabetic patient.

We next explored the variation of CCG profiles in Italian subjects, also in association with the dietary pattern and health conditions. To this aim, the dataset of analyzed individuals was integrated with publicly available16S rRNA gene sequences from 40 Italian obese patients suffering from type 2 diabetes and consuming a high-fat low-MACs diet (Candela et al., 2016). The addition of these samples did not result in an increase in the size of the Italian pan-microbiome, which still consisted of the previously identified 98 species. To compare the CCG distribution among individual gut microbiomes, for each CCG, the sum of the abundances of constituent microorganisms was calculated, and the personal profile of CCG abundance in the GM was computed for each subject. According to a clustering analysis of the individual CCG abundance profiles, subjects could be clustered into four groups (*p*-value < 0.001, permutation test with pseudo *F* ratio), each including individuals that shared a comparable profile of the four CCGs, and therefore potentially a similar carbohydrate-degrading functional pattern. We defined these redundant patterns as CAZyTypes (CTs), i.e., clusters of different GM configurations with a similar carbohydrate-degrading profile (**Figure 3**). Interestingly, CT2 and CT4 were more represented in healthy individuals consuming a Mediterranean diet, while CT1 and CT3 occurred with higher frequency in obese T2D patients consuming a high-fat low-MACs diet. By Principal Coordinates Analysis (PCoA), we represented the CCG variation patterns in individual microbiomes in a two-dimensional space (Supplementary Figure S2), highlighting the separation of subjects based on both the CCG abundance and health status (*p*-value < 0.001 permutation test with pseudo *F* ratio).

Looking at the CCG distribution in the Italian population, we found that the most prevalent CCG was *F. prausnitzii* group, with the co-presence of *E. rectale* group as an ancillary CCG in the health-prevalent CT2 and CT4 (**Figure 3**). On the other hand, *S. variabile* group and *R. bromii* group were sporadically represented in the subjects of all CTs, but generally present in lower abundance in healthy subjects. Indeed, only three healthy subjects out of 16 showed a relative abundance of the *S. variabile* and *R. bromii* groups higher than the average contribution in the overall dataset. Conversely, a higher abundance of these two CCGs was found in T2D patients (18 out of 40).

## Discussion

Our work aimed at characterizing the frame of the CAZyme variation in the GM meta-community from 56 Italian subjects, of whom 16 were healthy lean adults consuming a standard Mediterranean diet (Schnorr et al., 2014) and 40 were obese T2D patients consuming a high-fat low-MACs diet (Candela et al., 2016). The pan-microbiome of the studied population included a total of 98 bacterial species and, in healthy subjects, it was dominated by *F. prausnitzii*, *E. rectale*, *R. bromii* and *B. adolescentis*, which also emerged as the most prevalent species in the GM from Italian healthy adults. When compared with previously characterized GM pan-genomes, such as that from the Chinese population (Zhang et al., 2015), the Italian one showed some peculiarities, i.e., the presence of *E. rectale* and *Bifidobacterium* and the absence of *Phascolarctobacterium* within the core community. Although both studies are based on relatively small cohorts, and a more extensive screening at the population level is needed, these data seem to suggest a certain level of country specificity in the gut microbiome structure, which may contribute to the immunological and metabolic peculiarities of the populations.

According to our findings, the bacterial species belonging to the Italian pan-microbiome showed two different types of MAC-degrading profiles, essentially characterized by a high or low content of glycosyl-hydrolase-coding sequences, respectively. As expected, the CAZyme distribution in the various species of the Italian GM was heterogeneous, and the absolute number of CAZymes was independent from the genome size. However, it should be mentioned that, for each identified species, the analysis of the CAZyme content was performed by using the type strain reference genome deposited in the NCBI database and therefore our classification was blind with respect to the possible strain-level functional variability in the CAZyme profile.

The GM species of the Italian pan-microbiome were successfully clustered into four CCGs according to the similarity of the CAZyme pattern: *S. variabile* (CCG1), *E. rectale* (CCG2), *R. bromii* (CCG3), and *F. prausnitzii* (CCG4). Interestingly, each of the identified CCGs was characterized by a peculiar structure in terms of CAZyme content. In particular, *F. prausnitzii* and *R. bromii* groups were the most enriched CCGs in terms of represented CAZyme functions, whereas *F. prausnitzii* and *E. rectale* groups were the most equipped CCGs in terms of CAZymes specifically involved in the breakdown of non-digestible carbohydrates and non-starch polysaccharides (i.e., xylans, pectins, and mannans). These observations suggest that the Italian pan-microbiome is diversified in at least four patterns of carbohydrate degradation, raising several open questions related to: (i) the major determinants of the co-evolutionary processes underlying this differentiation; (ii) the relative contribution of host genetics, lifestyle and diet as drivers of this functional convergence; (iii) the ultimate connections between the observed CCGs and the host metabolic phenotype.

When exploring the quantitative distribution of CCGs in the individual microbiota from all subjects analyzed, we observed four robust clusters of subjects sharing a similar CCG profile, termed from CT1 to CT4. In particular, the CT1 and CT3 clusters included CCG4 (*F. prausnitzii* group) as the most prevalent CCG, being present in all individuals, and CCG3 (*R. bromii* group) and CCG1 (*S. variabile* group) as less prevalent, ancillary, and generally mutually exclusive groups. Conversely, CT4 was dominated by both CCG4 (*F. prausnitzii* group) and CCG2 (*E. rectale* group), which equally shared the ecosystem. Finally, CT2 showed CCG2 (*E. rectale* group) as the most prevalent group and CCG4 (*F. prausnitzii* group) as ancillary and less prevalent group, except for three subjects that were dominated by CCG1 (*S. variabile* group). These observations are indicative of a different ecological behavior for the diverse CCGs. Indeed, while CCG4 (*F. prausnitzii* group) appears to co-exist with all other CCGs, CCG2 (*E. rectale* group) and the CCGs 1 (*S. variabile* group)/3 (*R. bromii* group) are mutually exclusive. Confirming this, none of the CTs showed the simultaneous presence of CCG2 and CCG1 and/or CCG3. Taken together, these data suggest that the GM-host co-evolution process has resulted in the establishment of four well-defined functional steady states, i.e., the four CTs, each determined by the CCG propensity to share the same gut environment, and each conferring to the host a specific pattern of CAZymes.

In order to explore possible associations of these CTs with the host diet and health, we explored their variation in Italian healthy adults consuming a Mediterranean diet and obese T2D patients consuming a high-fat low-MACs diet. Interestingly, according to our data, most healthy subjects belonged to the CTs 2 and 4, which were characterized by the simultaneous presence of CCG4 (*F. prausnitzii* group) and CCG2 (*E. rectale* group). Conversely, the great majority of obese T2D patients belonged to CT1 and CT3, where CCG2 (*E. rectale* group) was substituted by CCG3 (*R. bromii* group) and/or CCG1 (*S. variabile* group). Although caution is needed in interpreting results, the analysis presented here suggests that a high-fat low-MACs diet, in the context of metabolic deregulation, such as obesity and T2D, could force changes in the GM CTs, supporting the presence of CCG1 (*S. variabile* group) and/or CCG3 (*R. bromii* group) to the detriment of CCG2 (*E. rectale* group). Interestingly, compared to CCG1 and CCG3, the CCG2 showed higher levels of enzymes involved in the degradation of non-digestible carbohydrates and non-starch polysaccharides, which are indeed abundant MACs in the Mediterranean dietary regimen. Though preliminary, our data highlight a possible adaptive or maladaptive nature for each of the four CT steady states that describe the Italian pan-microbiome. Indeed, the steady states CTs 2 and 4, that were generally associated with healthy hosts, seem to be the result of an adaptive microbiome-host co-evolution process, in which the interplay between diet, gut microorganisms and the host can contribute to overall metabolic health. On the contrary, the CTs 1 and 3 that were associated with T2D and a high-fat low-MACs diet, may result from a maladaptive microbiome–host process, in which this type of diet has led to the selection of CT steady states able to contribute to metabolic and/or immunological deregulation. Supporting these hypotheses, patients suffering from Behcet’s syndrome, a systemic inflammatory condition, showed a specific GM functional dysbiosis accompanied by a decreased production of SCFAs (Consolandi et al., 2015). Although the CAZyme profile has not yet been explored in patients with this disease, we can speculate that the observed reduction in SCFA biosynthesis could be the result of a maladaptive transition to CT1 or CT3, which would diminish the GM potential to provide the host with essential metabolites, such as butyrate, crucial to support immunological health.

## Conclusion

Our findings highlighted the existence of specific and well-defined GM functional layouts (CAZyTypes, CTs) for what concerns the ecosystem capacity to metabolize MACs, and support the hypothesis that the human GM has the ability to reconfigure its own CAZyme functional layout in response to dietary changes, with possible implications for the host health and metabolic regulation.

## Author Contributions

SR and MS: conceived, designed and performed the analysis; SR, MS, MC, and ST: wrote the manuscript; EB, SQ, and PB: revised and edited the draft. All authors discussed the results, commented on the manuscript and approved the final version.

## Conflict of Interest Statement

The authors declare that the research was conducted in the absence of any commercial or financial relationships that could be construed as a potential conflict of interest.

## Footnotes

1 http://metagenomics.anl.gov/linkin.cgi?project=mgp8810

2 http://hmpdacc.org/HM16STR/healthy/

3 https://www.ncbi.nlm.nih.gov/genome/

4 https://github.com/carden24/Bioinformatics_scripts/blob/master/hmmscan-parser.sh

5 http://www.cazy.org

6 http://metagenomics.anl.gov/mgmain.html?mgpage=project&project=mgp17675

## Supplementary Material

TABLE S1 Prevalence data and average abundance of the identified 98 bacterial species in the dataset.
