# Supplementary material for: Variation of Carbohydrate-Active Enzyme Patterns in the Gut Microbiota of Italian Healthy Subjects and Type 2 Diabetes Patients
Source: Front Microbiol. 2017 Oct 24;8:2079. doi: 10.3389/fmicb.2017.02079 (PMC5660705; doi:10.3389/fmicb.2017.02079)
Supplement: Supplementary file 2 [file Image_1.PDF]

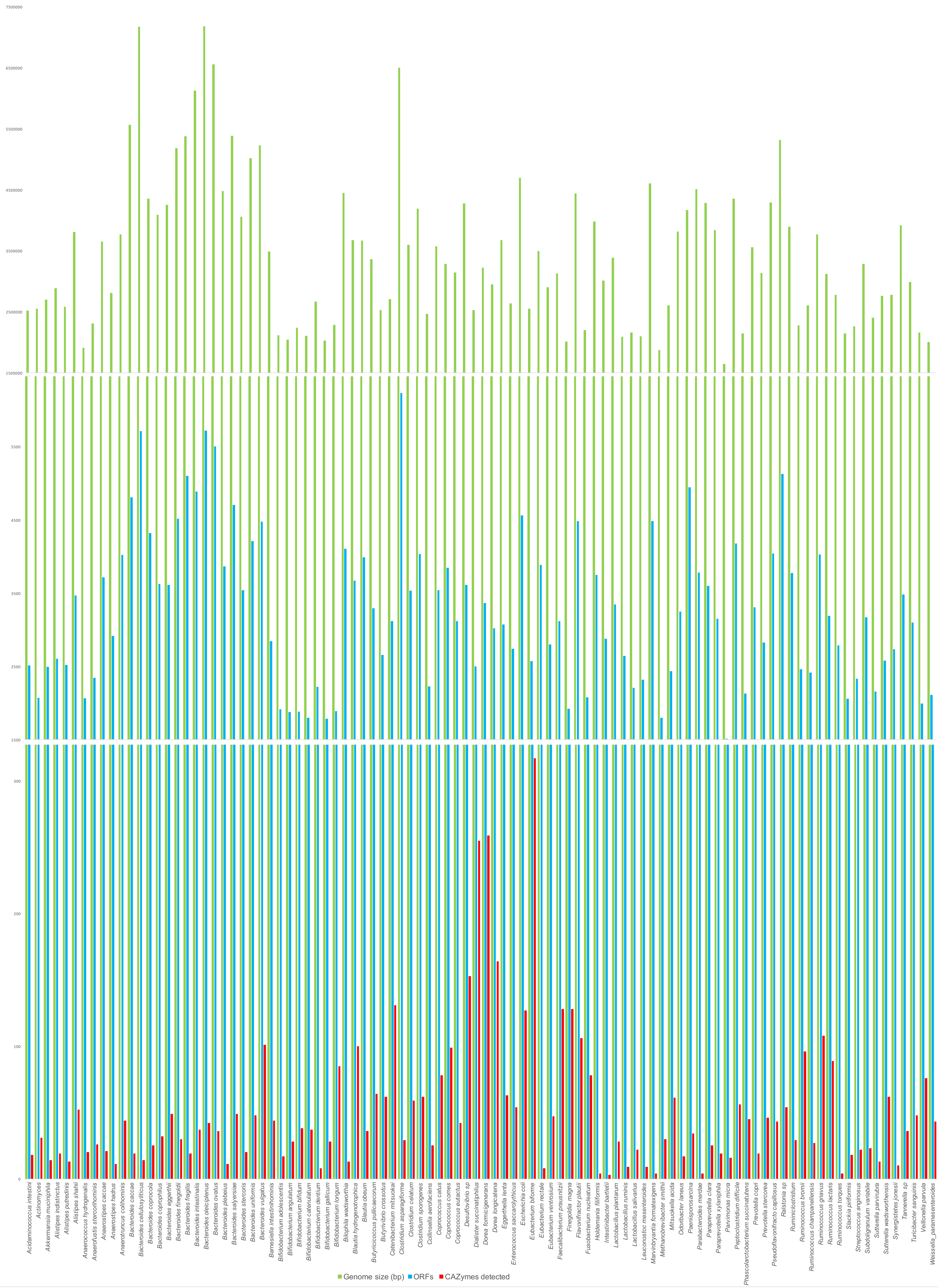

Supplementary Figure 1: Number of CAZyme-coding sequences detected in each analysed bacterial genome.
