# Supplementary material for: Variation of Carbohydrate-Active Enzyme Patterns in the Gut Microbiota of Italian Healthy Subjects and Type 2 Diabetes Patients
Source: Front Microbiol. 2017 Oct 24;8:2079. doi: 10.3389/fmicb.2017.02079 (PMC5660705; doi:10.3389/fmicb.2017.02079)
Supplement: Supplementary file 3 [file Image_2.PDF]

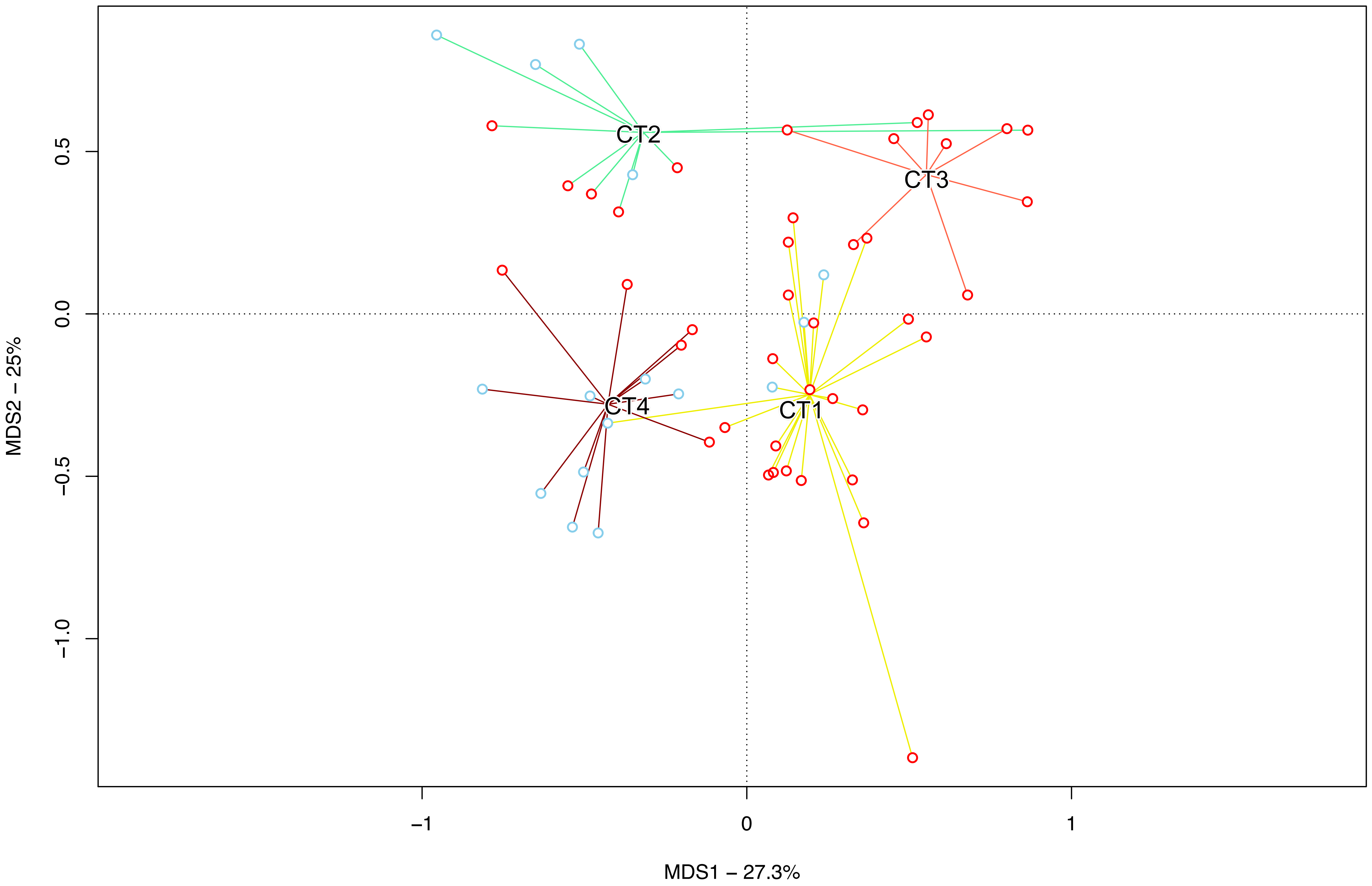

Supplementary Figure 2: PCoA based on Bray-Curtis distances between subjects taxonomical profiles, showing the CAZyType cluster distribution. Red dot, obese type 2 diabetic patient, blue dot, healthy lean subject (adonis permutational test, p value < 0.001).
